# Regulatory role of upstream AUG codons within the SIVmac239 genome

**DOI:** 10.1186/1742-4690-8-S2-P73

**Published:** 2011-10-03

**Authors:** Gisela J van der Velden, Atze T Das, Ben Berkhout

**Affiliations:** 1 Laboratory of Experimental Virology, Center for Infection and Immunity Amsterdam (CINIMA), Academic Medical Center, University of Amsterdam, The Netherlands

## 

Simian immunodeficiency virus from Rhesus macaques (SIVmac) is a primate lentivirus that exhibits extensive similarities with human immunodeficiency viruses (HIVs) in morphology, genome organization and biological properties. Like HIV, SIV is dependent on the host cellular machinery for transcription, translation and protein production. Alternative RNA splicing generates many mRNAs that allow the expression of all viral proteins. Consistent with the idea that translation occurs predominantly via a cap-dependent scanning mechanism, one usually does not find AUGs upstream of the open reading frames (ORFs) in HIV and SIV.

In SIVmac239, the envelope (Env) glycoprotein is translated from a 4 kb mRNA that also contains the upstream ORF (uORF) for Rev. It is currently accepted that the level of Env expression is dependent on leaky scanning due to suboptimal translation initiation at the upstream Rev AUG. Interestingly, another potential start codon is present immediately upstream of the Rev-AUG. We also identified an alternative Rev-Env mRNA that has in fact four upstream AUGs, raising questions about the regulation of Rev and Env translation. We constructed subgenomic Rev-Env reporter mRNAs to analyze how the different upstream AUGs affect protein expression. Our results indicate that the virus uses these unique upstream AUG codons to modulate the level of translation of the Rev and Env proteins.

